# HBx mediated Increase of SIRT1 Contributes to HBV-related Hepatocellular Carcinoma Tumorigenesis

**DOI:** 10.7150/ijms.43491

**Published:** 2020-07-09

**Authors:** Qing Wang, Sheng-tao Cheng, Juan Chen

**Affiliations:** Key Laboratory of Molecular Biology for Infectious Diseases, Chinese Ministry of Education, Chongqing Medical University, Chongqing, 400016, China.

**Keywords:** SIRT1, HBx, HCC, Metastasis.

## Abstract

**Objective:** Hepatocellular carcinoma (HCC) is one of the main causes of cancer-related deaths worldwide, and chronic hepatitis B virus (HBV) infection is strongly associated with HCC development, but the pathogenesis of HBV-related HCC remains obscure. Sirtuin 1 (SIRT1) has been implicated to enhance the replication of HBV and to promote the tumorigenesis of HCC. In this study, we aim to investigate the functional role of SIRT1 on HBV viral protein and HBV-induced HCC.

**Methods:** Tumorous liver tissues from patient diagnosed with HBV-related HCC were collected and further divided into two groups (with or without metastasis). Then, the mRNA and protein level of SIRT1 in those tissues were detected by real time PCR and Western blot, respectively. Meanwhile, the protein level of epithelial-mesenchymal transition (EMT) relative markers in those tissues was determined by Western blot. Furthermore, the expression of SIRT1 in HBV-expressing HCC cells was examined. Next, the relationship between viral proteins and SIRT1 expression were determined by real time PCR and Western blot. In addition, the potential role of HBx-upregulated SIRT1 in HCC proliferation, migration and invasion were analyzed by cell viability assays, cell proliferation assay, wound healing assay, transwell assay and Western blot.

**Results:** In this study, we found that the expression of SIRT1 was obviously increased in patients with metastasis compared to the patients without metastasis. Consistently, the expression of SIRT1 was also upregulated in HBV-expressing HCC cells compared to the controls. Further investigation showed that viral protein HBx was responsible for the elevated SIRT1 in HBV-expressing HCC cells. Meanwhile, the expression of HBx could be upregulated by SIRT1. Additionally, functional studies showed that HBx-elevated SIRT1 could promote HCC cell proliferation, migration and invasion. Importantly, HBx induced HCC proliferation and migration could be suppressed by Nicotinamide in a dose dependent manner.

**Conclusions:** Our findings uncovered the positive role of SIRT1 in HBx-mediated tumorigenesis which implicated the potential role of SIRT1 in HBV-related HCC treatment.

## Introduction

Sirtuins are the mammalian homologues of the yeast silent information regulator 2 (SIR2) 1 which are the highly conserved NAD^+^ -dependent histone deacetylases. Sirtuin 1 (SIRT1) is one of the early studied members of sirtuin family and is considered as a key regulator of various biological processes, especially in cancer. It has reported that SIRT1 is related to the tumorigenesis of gastric and colorectal cancers 2, the mitochondrial apoptosis of prostate cancer 3, and the prognosis of breast cancer 4. Importantly, SIRT1 is confirmed to be involved in the tumorigenesis 5, proliferation 6, metastasis 7, and chemical resistant 8 of HCC which hinted its potential role in liver cancer. As the major cause of HCC, HBV infection always receives much attention and there are increasing evidences to imply the functional role of SIRT1 in HBV replication. Our previous study displayed that SIRT1 can facilitate HBV replication by targeting the HBV core promoter which is mediated by transcriptional factor AP-1 9. Concordantly, Nicotinamide, the SIRT1 inhibitor, can inhibit HBV replication both *in vitro* and *in vivo* 10. Although extensive work has been carried out to elucidate the function of SIRT1 in HCC or HBV, little attention payed to the functional implications of SIRT1 on HBV-related HCC. Therefore, we focused on the role of SIRT1 on HBV-related HCC.

HBx is a protein encoded by the smallest ORF of HBV and act as an indispensable factor in initiating and maintaining HBV replication *in vivo* 11. As a key protein to regulate HBV replication, the role of HBx in the development and progression of liver cancer has also drawn great attention. Studies have found that HBx could regulate hepatocytes proliferation, regeneration and apoptosis through several signaling pathways, such as PI3K/Akt-mTOR signaling pathway 12, STAT3/Nanog signaling pathway 13 and Wnt/β-catenin signaling pathway 14 to promote the progression of HCC and other liver diseases. Based on the researches mentioned above, we can reach the agreement that HBx and SIRT1 indeed serve as the oncogenes in HCC, while the potential association between HBx and SIRT1 is still unclear. To investigate the underlined mechanism between HBx and SIRT1 in HBV-related HCC may provide a new understanding of the SIRT1 function.

The interaction between SIRT1 and HBV-mediated HCC has not been elucidated previously. In this study, we set out to explore the functional role of SIRT1 in HBV-related HCC. We found that viral protein HBx could upregulate SIRT1 expression and the hepatocarcinogenesis mediated by HBx is relied on the increased SIRT1. Our data implicated the positive role of SIRT1 in HBx-mediated tumorigenesis.

## Materials and methods

### Cell culture

Huh-7 cell line and HepG2 cell line were purchased from the HSRRB (Osaka, Japan) and ATCC (USA), respectively. Huh-7 and HepG2 cells were cultured in Dulbecco's modified Eagle medium (DMEM) (Corning, New York, USA) supplemented with 10% fetal bovine serum (FBS). Both HepG2.2.15 and HepAD38 cell lines were purchased from the Shanghai Second Military Medical University and cultured in DMEM containing 10% FBS, and 400 μg/ml of G418 (Merck, Germany). In addition, HepAD38 cells were grown in the presence of 0.3 μg/ml tetracycline. All the cells were maintained in a humidified incubator at 37°C with 5% CO2.

### Transfection

Transfection was carried out using Lipofectamine 3000^TM^ (Invitrogen, UAS). Cells were seeded into 12-well plates (HepAD38 and HepG2: 3×10^5^ cells per well; Huh-7: 2.5×10^5^ cells per well) 24h prior to the transfection and were grown to 70‑90% confluency prior to transfection. Specific operations are as follows: (1) Dilute Lipofectamine™ 3000 Reagent in Opti-MEM™ Medium. (2) Prepare master mix of DNA by diluting DNA in Opti-MEM™ Medium, and then add P3000™ Reagent. (3) Add Diluted DNA to each tube of Diluted Lipofectamine™ 3000 Reagent (1:1 ratio). (4) Incubate for 15 minutes at room temperature. (5) Add DNA-lipid complex to cells. After 6h of incubation, the supernatant was replaced with fresh medium. The transfection efficiency was monitored by Western blot analysis after 3 days of transfection.

### Plasmids and Antibodies

SIRT1 expression plasmid was constructed by in-frame insertion of the full-length SIRT1 into pcDNA3.1 which contains a Flag tag at the C terminus. Short hairpin RNA (shRNA) targeting SIRT1 (clone SH2421) or nontargeting shRNAs (clone SHC001) were purchased from Sigma-Aldrich. HBV expression plasmid pCH9/3091 was kindly provided by Prof. Lin Lan (The Third Military Medical University, Chongqing, China). HBx expression plasmid was constructed by in-frame insertion of the full-length HBx into p3×flag-CMV-7.1 which contains Flag tag at the C terminus. As described in our previous study 15, to construct HBV expression plasmid with HBx mutation (HBx MUT), a stop codon was inserted into the beginning of the HBx gene on pCH9/3091 (as the wild-type HBV, HBV WT).

Rabbit anti-SIRT1 monoclonal antibody was purchased from Cell Signaling Technology (2496, CST, MA, USA); Mouse anti-GAPDH monoclonal antibody was obtained from Zhongshan Golden Brige Biotechnology (TA-08, ZSGB-Bio, China); Rabbit anti-DDDDK tag Polyclonal Antibody (20543-1-AP), rabbit anti-E-Cadherin polyclonal antibody (20874-1-AP), rabbit anti-N-Cadherin polyclonal antibody (22018-1-AP) and rabbit anti-Vimentin polyclonal antibody (10366-1-AP) were purchased from Proteintech Group (Proteintech, Chicago, USA ).

### Western Blot

Cell or tissue proteins were lysed by RIPA lysis buffer with protease inhibitor (Roche, Mannheim, Germany). The concentration of total protein was determined by BCA (Roche, Mannheim, Germany) and the lysates containing 30 μg of total protein was separated by SDS-PAGE and the separated protein was transferred to nitrocellulose membrane (GE Healthcare, Buckinghamshire, UK), then incubated with indicated primary antibody (Anti-SIRT1 1:1000; Anti-GAPDH 1:10000; Anti-E-cadherin 1:5000; Anti-N-cadherin 1:2000; Anti-Vimentin 1:2000; Anti-DDDDK 1:3000) overnight at 4°C. The corresponding HRP-conjugated secondary antibody was further incubated at room temperature for 2 hours. Blots were developed by ECL Western blot reagents (Millipore, Massachusetts, USA). GAPDH was used as a loading control.

### Real-time PCR

TRIzol (TIANGEN, Beijing, China) methods were used to extract total RNA. IScript™ cDNA Synthesis Kit was acquired from Bio-Rad (Bio-Rad, California, USA). Relative expression level of SIRT1 was detected by Fast Start Universal SYBR Green Master Mix. β-actin mRNA was used as an endogenous control. The fold changes of various treatments were calculated by using the 2^-△△CT^ method. The sequences of primers are as follows: SIRT1, forward, CTAACTGGAGCTGGGGTGTCT, reverse, AAGTCTACAGCAAGGCGAGC.

### Cell Viability Assays

The cytotoxic effects of Nicotinamide on various cells were assessed by (3-(4,5-dimethylthiazol-2-yl)-2,5-diphenyltetrazolium bromide (MTT) assay (Sangon Biotech). Briefly, cells were seeded into 96-well plates (HepAD38 and HepG2: 2×10^4^ cells per well; Huh-7: 1.5×10^4^ cells per well) and treated with various concentrations of Nicotinamide (multiple proportion dilution from 128mM to 2mM) for 72 h. Next, 10μl MTT reagent (5mg/ml) were added into cell culture media and incubated for 4 h under the condition of protection from light. After that, dimethyl sulfoxide (DMSO) (Solarbio) was added to solubilize formazan dissolving in media. Finally, the absorbance at 490nm was recorded by using a 96-well plate reader. Fifty-percent cytotoxicity concentrations (CC50) were calculated via non-linear regression.

### Cell Proliferation Assays

Cell proliferation in response to different treatment was determined by trypan blue exclusion assay. Briefly, the cell suspension was mixed with a 0.4% trypan blue solution at a ratio of 9:1, and the dead cells were stained to blue while the viable cells were clear and colorless. The numbers of living cells were counted under a microscope.

### Wound-Healing Assay

Cell migration ability was measured by wound‑healing assay. Briefly, cells were seeded into 6-well plates (HepG2: 6×10^5^ cells per well; Huh-7: 5×10^5^ cells per well) and incubated at 37˚C. When cells reached 95-100% confluence, 10μl pipette tip was used to scratch the Monolayer cell. Cellular debris was removed by washing with PBS three times. The images of wound areas were captured with microscope three times (0, 24 and 48h) at a magnification of ×100.

### Transwell Migration and Invasion Assays

Cell metastasis ability was assessed by the transwell migration and invasion assay. 8×10^4^ cells and 1×10^5^ cells with indicated treatments were seeded into the transwell, respectively. After fixed by methanol at room temperature for 10 minutes, the cells migrated or invaded to the underside of the membrane were stained with 0.1% crystal violet at room temperature for 30 minutes and enumerated for 10 microscope fields.

### Patients Selection

With informed consent from patients, tumorous liver tissues were collected from 10 patients who underwent curative surgery for HCC at The First Affiliated Hospital of Chongqing Medical University. The 10 patients included 5 cases with metastasis and 5 cases without metastasis. All patients were hepatitis B virus s antigen (HBsAg) positive and diagnosed as HBV-related HCC. The research was approved by The Clinical Research Ethics Committee of the Chongqing Medical University.

### Statistical Analysis

Data are presented as the mean ± standard deviation (SD) from three independent experiments. Difference between two groups was assessed using two-tailed unpaired Student's t-test. One-way analysis of variance and two-way analysis of variance were performed for comparisons among multiple groups. All statistical analyses were performed by the SPSS version 19.0 software. A difference was considered significant when *P*<0.05.

## Results

### Relationship between SIRT1 expression and metastasis in HBV-related HCC patients

**Figure 1:**
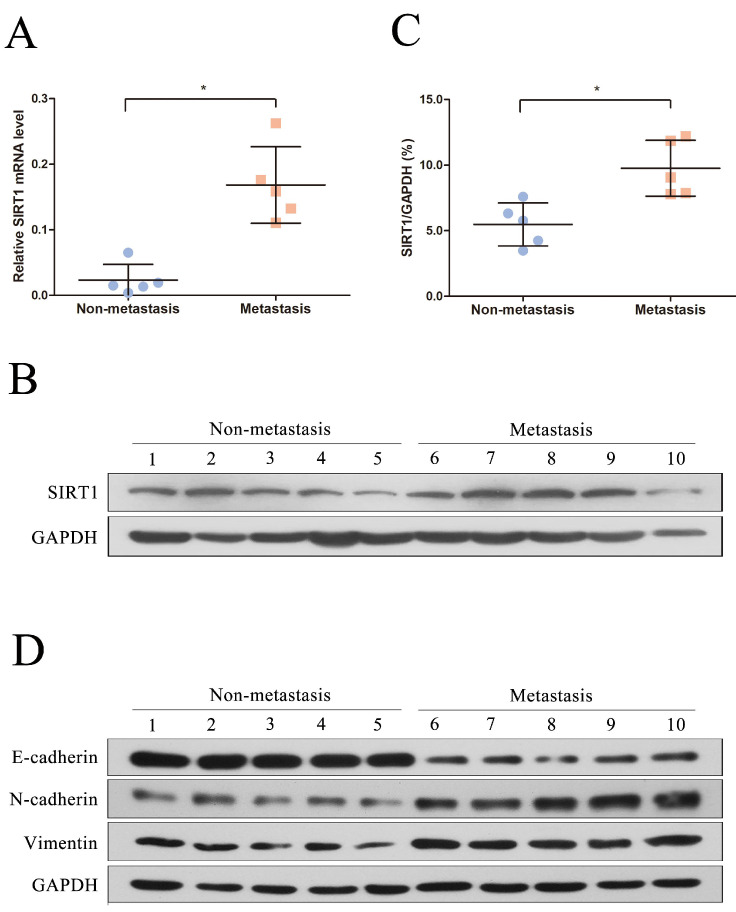
** SIRT1 corelated with the HCC metastasis in HBV-related HCC patients.** (A) Quantification analysis of SIRT1 mRNA level in HBV-related HCC tissue samples. The total RNA from liver tissues were extracted and determined by real-time PCR, β-actin was used as the internal controls. The central line represents the mean and the error bars indicate the standard deviation. Two-tailed unpaired Student's t-test was used to compare the groups. *p<0.05 (p=0.0079). (B) Total protein was extracted and the protein level of SIRT1 in HBV-related HCC tissue samples were examined by Western blot, GAPDH was used as loading controls. (C) Quantification analysis of SIRT1 protein level in HBV-related HCC tissue samples by Image J software. The central line represents the mean and the error bars indicate the standard deviation. Two-tailed unpaired Student's t-test was used to compare the groups. *p<0.05 (p=0.0079). (D) Total protein was extracted and the protein level of EMT-related markers (E-cadherin, N-cadherin and Vimentin) in HBV-related HCC tissue samples were examined by Western blot. GAPDH was used as loading controls.

We first recruited 10 patients diagnosed as HBV-related HCC in this study and the clinical and virological characteristics of the subjects were summarized in Table 1. The patients were further divided into two groups based on whether metastasis (metastasis group and non-metastasis group). To explore the potential role of SIRT1 in HBV-related HCC, the total RNA and protein were extracted and further subjected to real time PCR and Western blot. Compared with the non-metastasis group, the mRNA level of SIRT1 in metastasis group was upregulated significantly (Figure 1A). Concordantly, the protein expression level of SIRT1 in metastasis group was increased relative to non-metastasis group (Figure 1B-C), implied the positive role of SIRT1 in HBV-mediated HCC metastasis.

To further analyze the relationship between SIRT1 and metastasis, we detected the EMT-related markers by Western blot. Compared with the non-metastasis group, the protein levels of mesenchymal markers (N-cadherin and Vimentin) were significantly upregulated in metastasis group, while the epithelial markers E-cadherin was reduced (Figure 1D). Taken together, the above data hinted that SIRT1 may play a role in HBV-mediated HCC metastasis.

### SIRT1 expression is upregulated in HBV-expressing HCC cells

**Figure 2:**
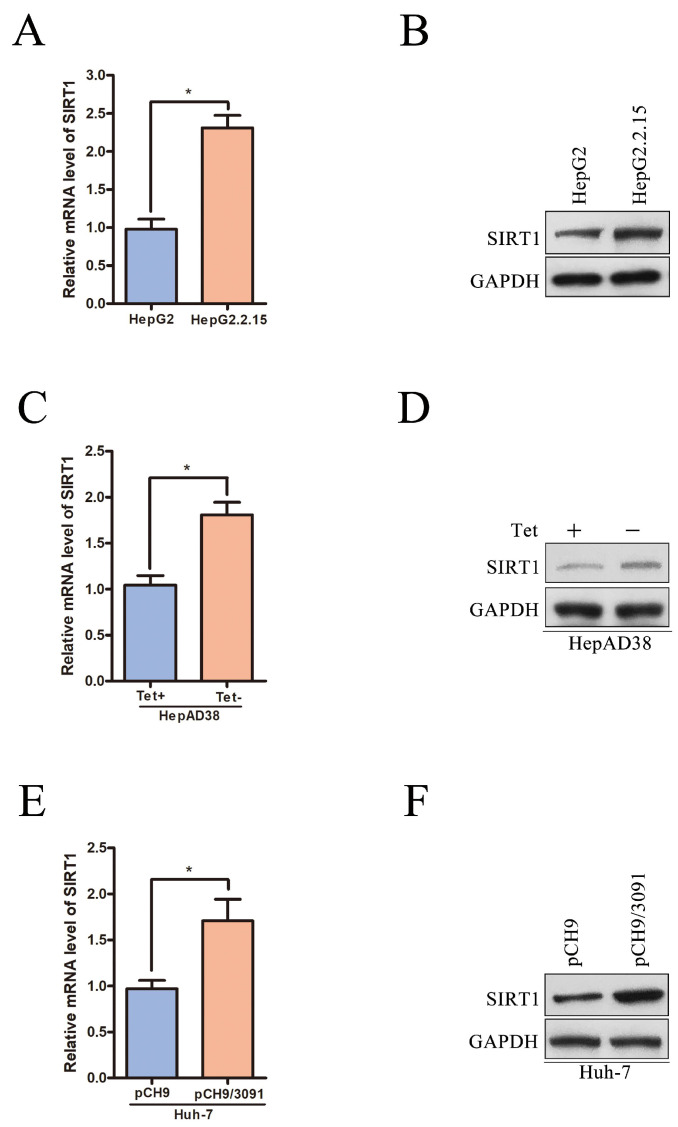
** SIRT1 is upregulated in HBV-expressing HCC cells.** (A-B) The mRNA and protein levels of SIRT1 in HepG2 and HeG2.2.15 cells were determined by real-time PCR and Western blot. β-actin and GAPDH were used as the internal controls, respectively. The data are presented as the mean ± standard deviation of 3 independent experiments, two-tailed unpaired Student's t-test was used to compare the groups. *p<0.05. (C-D) The mRNA and protein levels of SIRT1 in HepAD38 cells with or without tetracycline treatment were detected by real-time PCR and Western blot. β-actin and GAPDH were used as the internal controls, respectively. The data are presented as the mean ± standard deviation of 3 independent experiments, two-tailed unpaired Student's t-test was used to compare the groups. *p<0.05. Tet: tetracycline. (E-F) The HBV expressing plasmid pCH9/3091 or control vector was transfected into Huh-7 cells. Both mRNA and protein levels of SIRT1 were examined by real-time PCR and Western blot. β-actin and GAPDH were used as the internal controls, respectively. The data are presented as the mean ± standard deviation of 3 independent experiments, two-tailed unpaired Student's t-test was used to compare the groups. *p<0.05.

To further certificate the relationship between SIRT1 and HBV, we examined the expression level of SIRT1 in a panel of HBV expressing HCC cells. The mRNA and protein levels of SIRT1 were first compared in HepG2 and HepG2.2.15 cell lines. HepG2 is the parental cell line of HepG2.2.15 and HepG2.2.15 is an HBV stably transfected cell line constitutively producing HBV. We found that both the mRNA and protein levels of SIRT1 in HepG2.2.15 cells were increased compared to those in HepG2 cells (Figure 2A-B). Considering that the different cell lines may interfere the gene expression profile, the mRNA and protein levels of SIRT1 were further examined in HepAD38 cell lines which is an HBV stably transfected cell line to produce HBV continuously under the control of tetracycline. The data showed that the mRNA and protein levels of SIRT1 were upregulated in HepAD38 cells without tetracycline treatment (HBV production) relative to the cells with tetracycline treatment (no HBV production) (Figure 2C-D).

The expression level of SIRT1 was also detected in human hepatoma Huh-7 cells transiently transfected with HBV expressing plasmid pCH9/3091 (containing a 1.1-unit length HBV genome driven by a cytomegalovirus promoter). Compared with the cells transfected with vector, both mRNA and protein levels of SIRT1 were upregulated in cells transiently transfected with pCH9/3091 (Figure 2E-F). The above data fully proved that HBV could upregulate the SIRT1 expression.

### Positive interaction between SIRT1 and HBx in HCC cell lines

**Figure 3:**
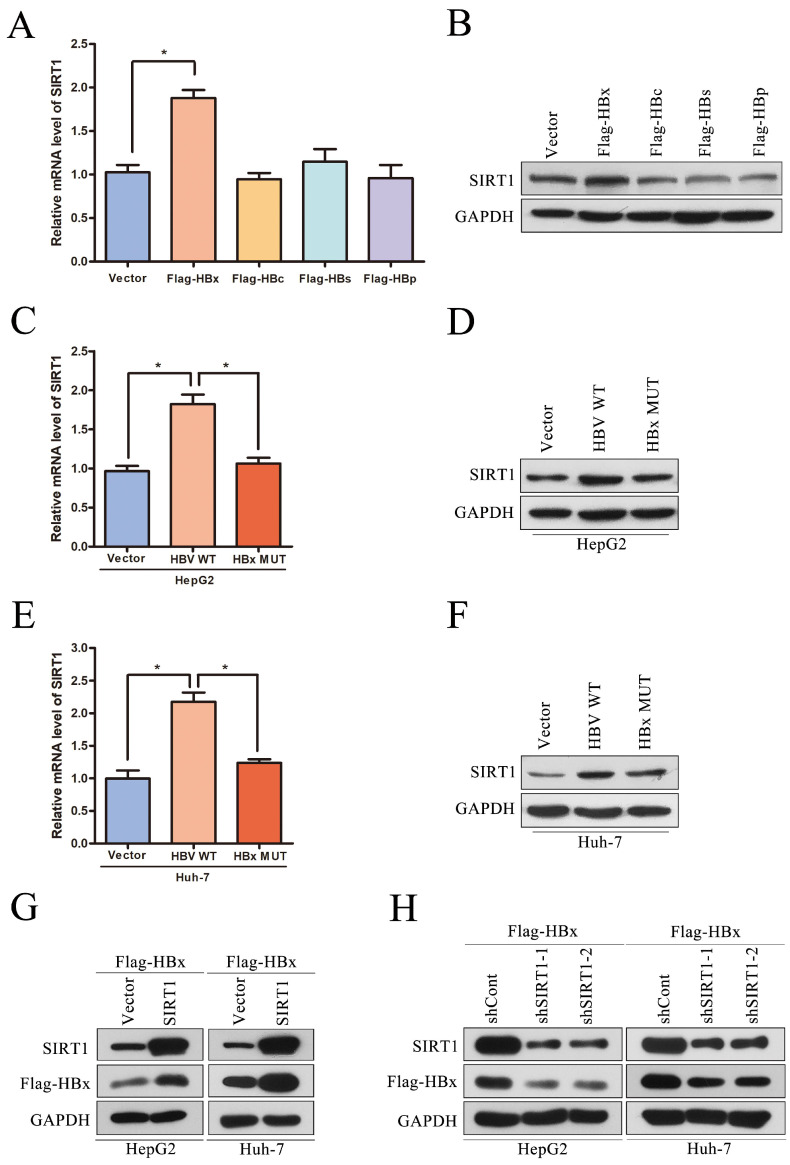
** Positive interaction between SIRT1 and HBx in HCC cell lines.** (A-B) The vector, Flag-HBx, Flag-HBc, Flag-HBs or Flag-HBp plasmids were transfected into Huh-7 cells. Both mRNA and protein levels of SIRT1 were examined by real-time PCR and Western blot. β-actin and GAPDH were used as the internal controls, respectively. The data are presented as the mean ± standard deviation of 3 independent experiments and the groups were compared using one-way analysis of variance. *p<0.05. (C-F) The vector, HBV WT or HBx MUT plasmids were transfected into HepG2 and Huh-7 cells. Both mRNA and protein levels of SIRT1 were examined by real-time PCR and Western blot. β-actin and GAPDH were used as the internal controls, respectively. The data are presented as the mean ± standard deviation of 3 independent experiments and the groups were compared using one-way analysis of variance. *p<0.05. (G) The vector, the SIRT1 overexpression plasmid were cotransfected with Flag-HBx into HepG2 and Huh-7 cells. The protein level of SIRT1 and HBx were detected by Western blot, GAPDH was used as loading controls. (H) The shCont, the shRNA targeting SIRT1 were cotransfected with Flag-HBx into the HepG2 and Huh-7 cells. The protein level of SIRT1 and HBx were detected by Western blot. GAPDH was used as loading controls.

Although we have reported that SIRT1 can promote HBV replication through AP-1 by targeting the HBV core promoter 9, the potential role of HBV on SIRT1 expression is still remained to be elucidated. The HBV genome has four open reading frames (ORFs) encoding the following proteins: X protein (HBx), preCore/core protein (HBc), S protein (HBs) and Pol protein (HBp). To determine which viral protein is responsible to the upregulation of SIRT1, the plasmids expressing the indicated viral proteins were transiently transfected into Huh-7 cells. The mRNA and protein levels of SIRT1 were examined by real-time PCR and Western blot, respectively. Obviously, ectopic expression of HBx could increase the expression level of SIRT1, while overexpression of HBc, HBs, or HBp had no significant effect on SIRT1 expression (Figure 3A-B).

To further confirm that HBx is responsible for the elevated SIRT1 in HBV expressing cells, HepG2 or Huh-7 cells were transfected with HBV expressing plasmid (HBV WT), HBV expressing plasmid with X gene abrogated (HBx MUT) or control plasmid (Vector). As expected, HBx mutation abolished the SIRT1 upregulation induced by wild type HBV in both two cell lines (Figure 3C-F), which indicated that HBx could upregulate SIRT1 expression. We further studied the functional role of SIRT1 in HBx expression. As expected, the expression of HBx could be upregulated by SIRT1 overexpression (Figure 3G) while downregulated by SIRT1 depletion (Figure 3H). Taken together, there is a positive interaction between SIRT1 and HBx.

### Functional role of SIRT1 overexpressing in HBx-mediated HCC cell proliferation, migration and invasion

**Figure 4:**
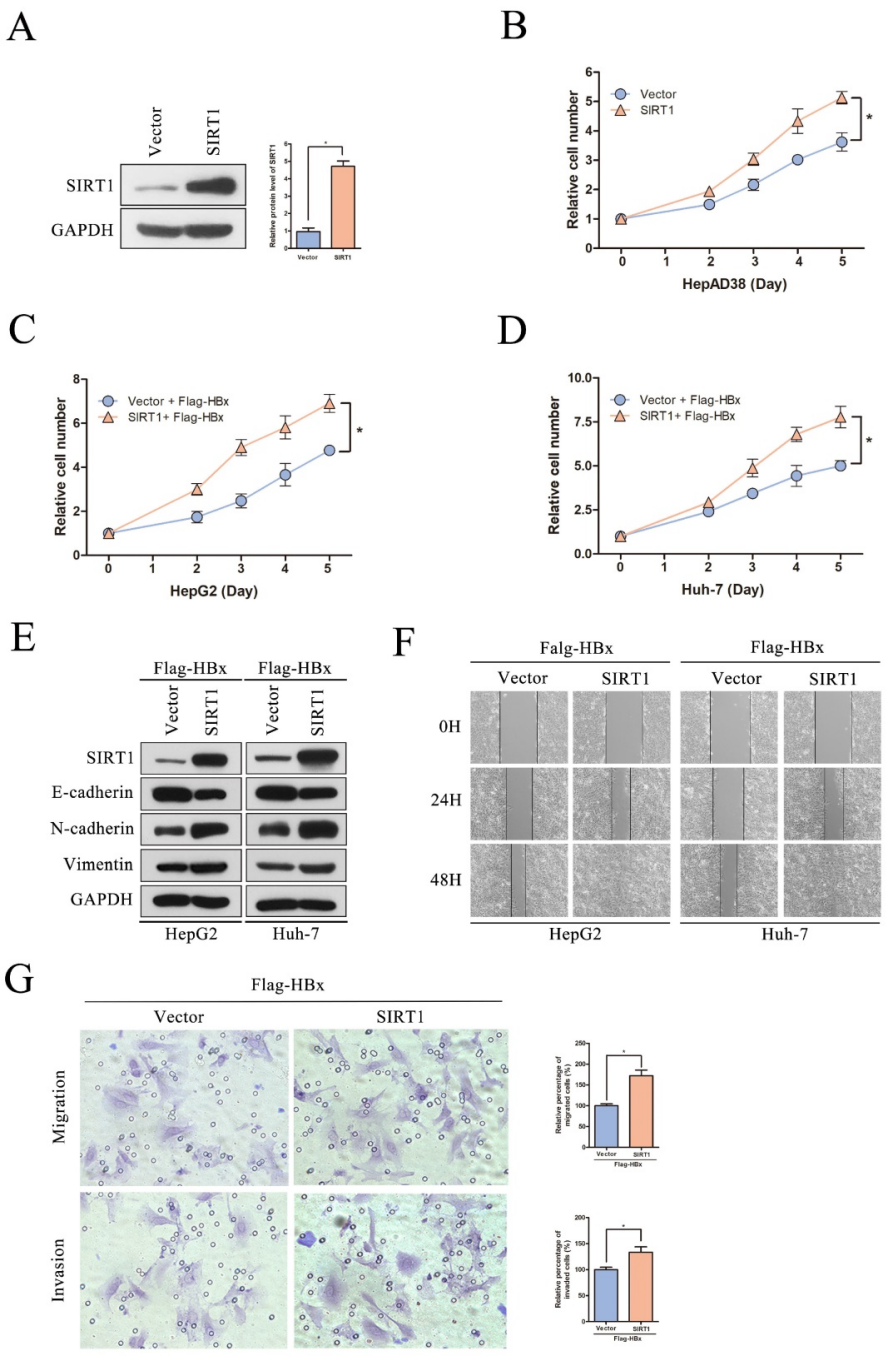
** The overexpression of SIRT1 could promote HBx-mediated HCC cell proliferation, migration and invasion.** (A) The SIRT1 protein level was detected by Western blot. GAPDH was used as loading controls. Quantification of SIRT1 protein level were analyzed by Image J software. The data are presented as the mean ± standard deviation of 3 independent experiments, two-tailed unpaired Student's t-test was used to compare the groups. *p<0.05. (B) The overexpression of SIRT1 could promote HepAD38 cell growth. The data are presented as the mean ± standard deviation of 3 independent experiments and the groups were compared using two-way analysis of variance. *p<0.05. (C-D) The SIRT1 overexpression plasmid were cotransfected with Flag-HBx into the in HepG2 and Huh-7 cells and the cell numbers at indicated time points were recorded. The data are presented as the mean ± standard deviation of 3 independent experiments and the groups were compared using two-way analysis of variance. *p<0.05. (E) Western blot exhibited the overexpression of SIRT1 could induce the expression of mesenchymal markers (N-cadherin and Vimentin) and reduce the expression of epithelial marker (E-cadherin) in HBx-mediated HepG2 and Huh-7 cells, GAPDH was used as loading controls. (F) Wound-healing assay (magnification, ×100) showed that SIRT1 overexpression could promote HBx mediated cell migration in HepG2 and Huh-7 cells. Representative images in each group are shown. (G) The overexpression of SIRT1 could promote HBx mediated cell migration and invasion in Huh-7 cells which determined by transwell migration and invasion assays, representative images in each group are shown. Migrated and invaded cells were counted and expressed as a percentage relative to the control group. The data are presented as the mean ± standard deviation of 3 independent experiments, and the groups were compared using two-tailed unpaired Student's t-test. *p<0.05.

It has reported that HBx could interplay with several signaling pathways associated with cell proliferation, migration and invasion 16. To investigate that whether HBx-elevated SIRT1 plays a role in HCC cell proliferation and metastasis, a series assays were conducted on cell models. The SIRT1 overexpressing plasmid was transfected in HepAD38 cells and the overexpression efficiency was first confirmed by Western blot (Figure 4A). And SIRT1 overexpression resulted in increased proliferation rate of HepAD38 cells (Figure 4B) which hinted that SIRT1 plays a role in HBx-mediated cell proliferation.

To further explore the functional role of SIRT1 in HBx-mediated HCC development, the SIRT1 overexpression plasmid was cotransfected with Flag-HBx into HepG2 and Huh-7 cells. Increased cell proliferation rates were observed in both two cell lines which suggested that HBx facilitates HepG2 and Huh-7 cells proliferation via a SIRT1 dependent way (Figure 4C-D). Then, the EMT-related markers were determined by Western blot and we found that SIRT1 could increase the protein level of mesenchymal markers (N-cadherin and Vimentin) and decrease the protein level of epithelial marker E-cadherin (Figure 4E). Meanwhile, SIRT1 overexpression could facilitate cell migration which witnessed by wound healing assay (Figure 4F). Consistently, HBx enhances Huh-7 cells migration and invasion depended on SIRT1 (Figure 4G). Undoubtedly, our results supported that the role of HBx in promoting HCC depended on SIRT1.

### Effect of SIRT1 silencing on HBx-mediated HCC cell proliferation, migration and invasion

**Figure 5:**
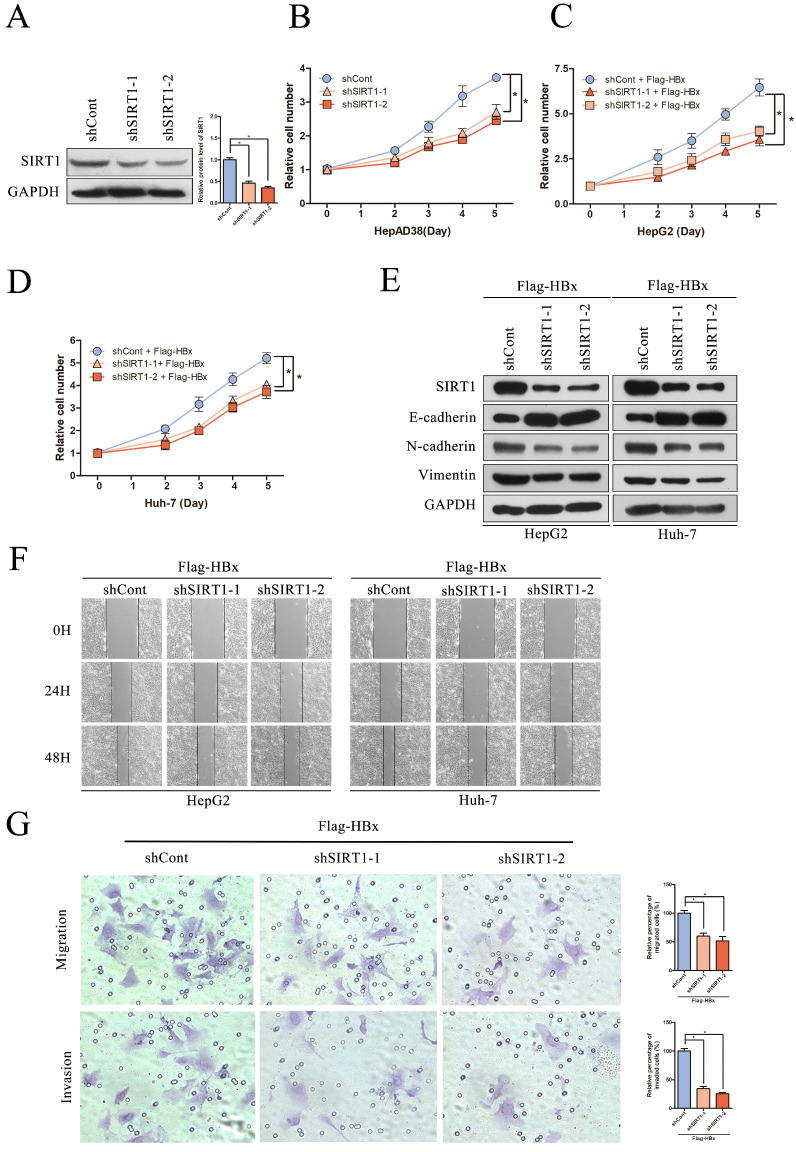
** The depletion of SIRT1 could inhibit HBx-mediated HCC cell proliferation, migration and invasion.** (A) The efficiency of SIRT1 gene silencing was evaluated by western blotting, GAPDH was used as loading controls. Quantification of SIRT1 protein level were analyzed by Image J software. The data are presented as the mean ± standard deviation of 3 independent experiments and the groups were compared using one-way analysis of variance. *p<0.05. (B) The depletion of SIRT1 could inhibit HepAD38 cell growth, the cells were counted at the indicated time points. The data are presented as the mean ± standard deviation of 3 independent experiments and the groups were compared using two-way analysis of variance. *p<0.05. (C-D) The shRNA targeting SIRT1 were cotransfected with Flag-HBx into the HepG2 and Huh-7 cells and the cell numbers at indicated time points were recorded. The data are presented as the mean ± standard deviation of 3 independent experiments and the groups were compared using two-way analysis of variance. *p<0.05. (E) The depletion of SIRT1 could induce the protein level of epithelial marker (E-cadherin) and reduce the expression of mesenchymal markers (N-cadherin and Vimentin) in HBx-mediated HepG2 and Huh-7 cells, which determined by Western blot, GAPDH was used as loading controls. (F) The depletion of SIRT1 could inhibit HBx mediated cell migration in Huh-7 and HepG2 cells which determined by wound healing assay (magnification, ×100). Representative images in each group are shown. (G) The depletion of SIRT1 could inhibit HBx mediated cell migration and invasion in Huh-7 cells which were examined by transwell migration and invasion assays, and representative images in each group are shown. Migrated and invaded cells were counted and expressed as a percentage relative to the control group. The data are presented as the mean ± standard deviation of 3 independent experiments and the groups were compared using one-way analysis of variance. *p<0.05.

To evaluate the significance of SIRT1 in hepatocarcinogenesis mediated by HBV, the SIRT1 short hairpin RNAs (shSIRT1-1 and shSIRT1-2) were introduced into HepAD38 cells. As evidenced by Western blot, shSIRT1-1 and shSIRT1-2 downregulated the SIRT1 expression effectively (Figure 5A). As shown in Figure 5B, depletion of SIRT1 significant inhibited the proliferation of HepAD38 cells compared to the control group (Figure 5B).

To gain a better understanding of the association of HBx-elevated SIRT1 on HCC cell proliferation, shRNAs targeting SIRT1 were cotransfected with Flag-HBx into the HCC cells and the cell numbers were counted at indicated time points. In contrast to SIRT1 overexpression, SIRT1 silencing resulted in decreased cell proliferation in both HepG2 and Huh-7 cells (Figure 5C-D). Additionally, the increased E-cadherin was observed in SIRT1 silencing cells, as well as decreased N-cadherin and Vimentin (Figure 5E). Similarly, wound healing assay revealed that SIRT1 depletion abolished the effect of HBx on cell migration (Figure 5F). Furthermore, transwell assay also confirmed that SIRT1 depletion abolished the capacity to migrate and invade in Huh-7 cell (Figure 5G). Those data further demonstrated that HBx promoted hepatocarcinogenesis through a SIRT1 dependent way.

### Effect of Nicotinamide on HBx-mediated HCC cell proliferation and migration

**Figure 6:**
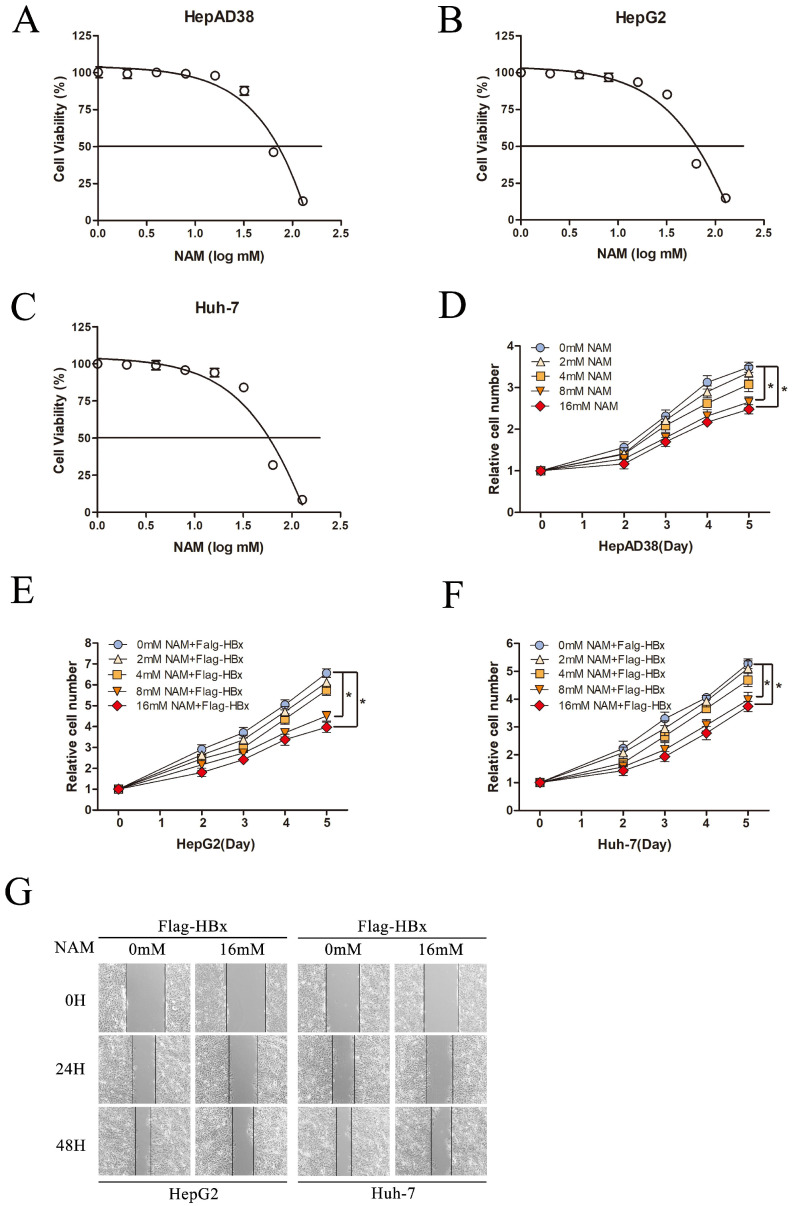
** Inhibition SIRT1 could inhibit HBx-mediated HCC cell proliferation and migration.** (A-C) Cytotoxic effects of Nicotinamide in the HepAD38, HepG2 and Huh-7 cell lines. Cell viability was measured via MTT assays. The CC50 value of Nicotinamide was 58.3 mM in HepAD38 cells, 52.9 mM in HepG2 cells and 55.7 mM in Huh-7 cells. (D) Nicotinamide could inhibit HepAD38 cell growth in a dose dependent manner, the cells were counted at the indicated time points. The data are presented as the mean ± standard deviation of 3 independent experiments and the groups were compared using two-way analysis of variance. (E-F) Nicotinamide resulted in decreased proliferation rate in HBx-mediated HepG2 and Huh-7 cells in a dose dependent manner, the cell numbers at indicated time points were recorded. The data are presented as the mean ± standard deviation of 3 independent experiments and the groups were compared using two-way analysis of variance. (G) Wound healing assay (magnification, ×100) showed that 16mM Nicotinamide resulted in effective inhibition of HBx mediated cell migration in HepG2 and Huh-7 cells. Representative images in each group are shown. NAM: Nicotinamide.

To fully understand the functional role of SIRT1 in HBx-induced HCC, we next explored the effect of SIRT1 inhibitor, Nicotinamide on hepatocarcinogenesis in our study. Cell cytotoxicity of Nicotinamide was first determined by using MTT assay. Nicotinamide exhibited cell toxicity at concentrations higher than 16mM. And CC50 were 58.3mM in HepAD38 cells, 52.9 mM in HepG2 cells and 55.7mM in Huh-7 cells (Figure 6A-C).

To investigate the potential effect of Nicotinamide on hepatocarcinogenesis mediated by HBV, HepAD38 cells were treated with various concentrations of Nicotinamide as indicated. The data showed that Nicotinamide could inhibit cell proliferation in a dose dependent manner (Figure 6D). More importantly, Nicotinamide could also suppress HBx-expressing cells proliferation of in a dose dependent manner (Figure 6E-F). Also, the impact of Nicotinamide on HBx-mediated HCC cell migration was detected by wound healing assay. The data showed that cell migration was obviously inhibited by Nicotinamide (Figure 6G). Unquestionable, our results displayed that inhibition SIRT1 could inhibit HBx-mediated HCC cell proliferation and migration, which implied the positive function of SIRT1 inhibitor Nicotinamide in HBV-related HCC treatment.

## Discussion

We identified that SIRT1 is elevated in HBV-related HCC patients with metastasis by detected the expression of SIRT1 in tumor tissues from the patients. As EMT is an extremely important step in cancer metastasis, we then detected the protein level of EMT-related markers in those tissues. Consistently, mesenchymal markers N-cadherin and Vimentin were upregulated and epithelial markers E-cadherin was reduced in metastasis group. Interestingly, the expression of SIRT1 is positively correlated with mesenchymal markers. This finding intensively indicates the functional role of SIRT1 in HBV-related HCC metastasis which hinted that inhibition of SIRT1 may be the new strategy for HBV-related HCC treatment. In line with our study, Portmann S, *et al.* states that SIRT1 inhibition have the antitumor effect in human HCC tumor models *in vitro* and *in vivo* 18. Recently, a report shows that the mutation of SIRT1 led to a decreased risk of HCC 25 reminds us that the complete function of SIRT1 is necessary to promote cancer progression. This study also reveals that interfering the function of SIRT1 by effective mutation may be a new method to inhibit HCC.

Several groups have reported that SIRT1 is involved in the oncogenesis of HCC 7, 17, 18 and the regulation of HBV replication 19 which promoted us to investigate the role of SIRT1 in HBV-related HCC. In contrast to the inhibitory effect of SIRT1 inhibitor on HBV replication 10, SIRT1 overexpression is benefit to the transcription and replication of HBV 9. While the above two studies only demonstrate the effect of SIRT1 on HBV, our results further display the positive effect of HBV or HBx on SIRT1. In this study, we found that the mRNA and protein level of SIRT1 could be upregulated by HBx in HCC cells by transfected with wild type HBV expressing plasmid or HBV expressing plasmid with HBx gene mutation. Meanwhile, HBx could be upregulated by SIRT1 in HBx-mediated HCC cells by transfected with SIRT1 overexpressing plasmid or shRNAs targeting SIRT1. Coincidence with our findings that there is a positive interaction between SIRT1 and HBx, Deng JJ, *et al.* reports that the protein level of SIRT1 is upregulated in HBV-replicating hepatoma cells. Furthermore, SIRT1 could promote HBV transcription by promoting the recruitment of HBx on covalently closed circular DNA (cccDNA), leading to the robust production of cccDNA 20. It is known that HBx is a general activator of gene expression 21, this reminds us that HBx may elevate the SIRT1 level by targeting its promoter to enhance the transcriptional activity of SIRT1. However, detailed mechanism is not defined in this study. Further researches for the underlying mechanism of HBx upregulates the expression of SIRT1 in HBV-related HCC are necessary.

Based on our study, the promoted effect of HBx on hepatocarcinogenesis is depending on SIRT1. The previous studies have elucidated many potential mechanisms about the HBx to facilitate HCC development. A mechanistic study demonstrates that HBx activated AKT may augment the dedifferentiation of hepatocytes to promote HCC progression 22. Also, a recent study points out that HBx mutations can enhance HCC migration through the Wnt/β-catenin signaling pathway 14. Importantly, Kim HY, *et al.* holds that downregulation of HBx could overcome the chemoresistance to sorafenib *in vitro* 23. However, except for the above mechanisms, we believe that HBx participates in the HCC cell proliferation, migration and invasion via a SIRT1 dependent way. It is worth noting that SIRT1 overexpression significantly enhanced cell proliferation, migration and invasion in HBx-mediated HCC cells, which reveals the important role of SIRT1 in HBx-mediated HCC tumorigenesis. Meanwhile, we found that SIRT1 could induce the expression of mesenchymal markers and reduce the expression of epithelial markers, which suggest that the activation of EMT is probably involved in the underlining mechanism of SIRT1 promotes HBx-mediated HCC tumorigenesis. As for the relationship between HBx and SIRT1, it has reported that HBx can attenuate the interaction between SIRT1 and β-catenin, which leads to the resistance to chemotherapy drugs 24. As SIRT1 involved in the multiple biological process of HBx-mediated biological process of HCC, the investigation about SIRT1 and HBx could deepen the understanding of interaction between those two factors and shed a light on HBV-related HCC treatment.

In general, our work suggests that SIRT1 may serve as the mediate to promote hepatocarcinogenesis and inhibition of SIRT1 may block the tumor process mediated by HBx. Further studies are needed to determine whether SIRT1 could be exploited as a target for HBV-related HCC therapy and to assess Nicotinamide possible therapeutic applications in HBV-related HCC treatment.

## Figures and Tables

**Table 1:** Clinical and virological characteristics of the subjects enrolled in the study.

Patient	Age (y)	Gender (F/M)	HBV DNA (IU/ml)	HBsAg (IU/ml)	ALT (IU/ml)	Metastasis (Yes/No)	Differentiation grade
1	46	M	1.32×105	2492	39	Y	Moderate
2	50	M	2.87×106	1913.91	45	Y	Moderate
3	46	M	3.58×107	2254.55	29	Y	Well
4	43	M	5.5 ×105	1214	34	Y	Moderate
5	46	M	1.61×105	2018	80	Y	Moderate
6	53	M	1.46×106	1587	83	N	Moderate
7	40	M	3.52×106	2236	82	N	Well
8	47	M	1.1 ×106	1912	50	N	Moderate
9	43	M	2.53×107	243	51	N	Moderate
10	46	M	7.89×10^5^	20.33	35	N	Moderate

## Abbreviations

HCC: Hepatocellular carcinoma
HBV: Hepatitis B virus
NAM: Nicotinamide
EMT: epithelial-mesenchymal transition
SIRT1: Sirtuin 1
